# Unusual Spinal Epidural Lipomatosis and Lumbosacral Instability

**DOI:** 10.1155/2016/3094601

**Published:** 2016-03-16

**Authors:** David Ruiz Picazo, José Ramírez Villaescusa

**Affiliations:** Department of Orthopedic Surgery, Spine Surgery Unit, Complejo Hospitalario Universitario de Albacete, Universidad de Castilla La Mancha, Hermanos Falco 36, Albacete, 02006 Castilla La Mancha, Spain

## Abstract

*Introduction.* Epidural lipomatosis is most frequently observed in patients on chronic steroid treatment. Only a few idiopathic epidural lipomatosis cases have been described.* Material and Methods.* 64-year-old male patient presented with low back pain and left leg pain. Later, the patient experienced neurogenic claudication and radicular pain in the left leg without urinary dysfunction. Plain radiography and magnetic resonance imaging demonstrated an abnormal fat tissue overgrowth in the epidural space with compression of the dural sac, degenerative disc disease at L4-L5 level, and instability at L5-S1. Endocrinopathic diseases and chronic steroid therapy were excluded. If conservative treatment failed, surgical treatment can be indicated.* Results.* After surgery, there was a gradual improvement in symptoms and signs, and six months later the patient returned to daily activities and was neurologically normal.* Conclusion.* In the absence of common causes of neurogenic claudication, epidural lipomatosis should be considered. The standard test for the diagnosis of epidural lipomatosis is magnetic resonance (MR). At first, conservative treatment must be considered; weight loss and the suspension of prior corticosteroid therapy are indicated. In the presence of neurological impairment, the operative treatment of wide surgical decompression must be performed soon after diagnosis.

## 1. Introduction

Spinal epidural lipomatosis (SEL) is a pathological accumulation of fat in the epidural space of the vertebral canal. This pathology is specifically related to patients receiving corticosteroid therapy (e.g., transplant, systemic lupus erythematosus, dermatomyositis, rheumatoid arthritis, and chronic obstructive pulmonary disease patients) [1, 2]. SEL has also been described in patients with morbid obesity, Cushing's syndrome, hypothyroidism, bronchial carcinoma, and testis seminoma. However, idiopathic SEL is extremely rare compared to cases resulting from corticosteroid therapy. Usually, these patients present with lower back pain with variable neurological impairment (sensitivity and motor changes in the lower extremities). More uncommon signs of idiopathic SEL are bowel and bladder incontinence. The findings of examination vary depending on the location of the SEL, thoracic or lumbar (spinal cord, conus medullaris, and cauda equina). MRI is the test of choice and has replaced computed tomography (CT) scans and myelograms. The initial study should rule out other causes of lower back pain, such as degenerative disease and tumours. Treatment options of symptomatic SEL include exercise, weight reduction, and surgical decompression.

Here, we present a case of idiopathic SEL in a male patient with low back pain and left S1 radiculopathy (pain and paresthesias) that did not respond to conservative treatment, including medications and physical therapy. Moreover, the patient presented with L5-S1 instability secondary to low grade spondylolisthesis due to spondylolysis and degenerative disc disease at the L4-L5 level. He underwent decompression surgery and stabilization with circumferential and posterolateral fusion.

## 2. Case Report

**Figure 1:**
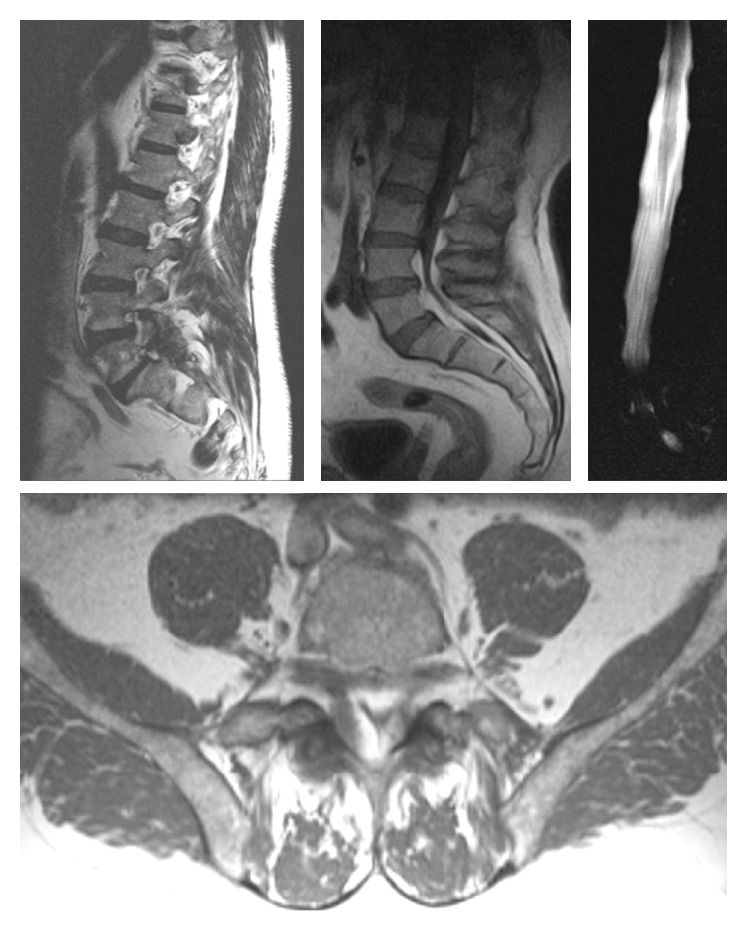
Preoperative MR. T1-weighted axial and sagittal image showed Y sign and thin dural sac. MR-myelogram shows stop at L4-S1 levels. Sagittal T2- (left) and T1- (right) weighted MR images showing Modic type I endplate change at the L4-L5 and L5-S1 levels consisting of low signal intensity on T1 images and high signal intensity on T2 images.

**Figure 2:**
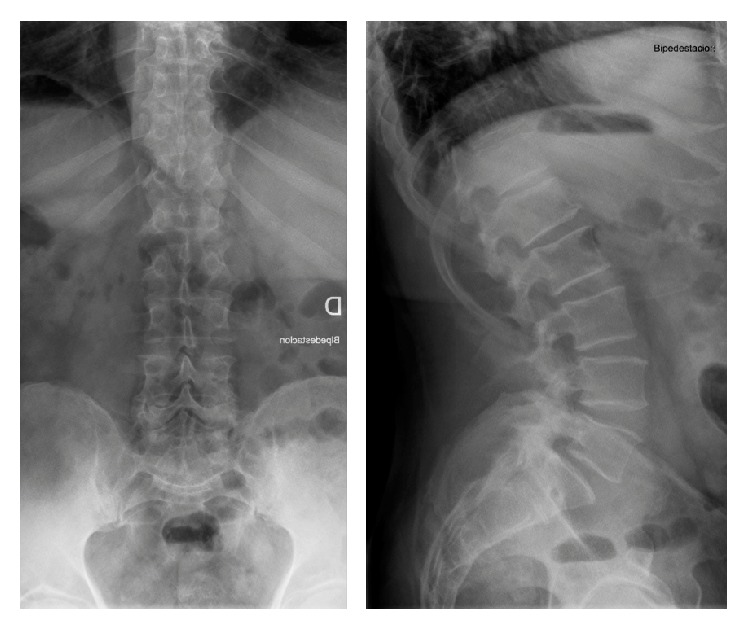
Preoperative anteroposterior and sagittal X-ray. Spondylolisthesis L5-S1 grade I.

**Figure 3:**
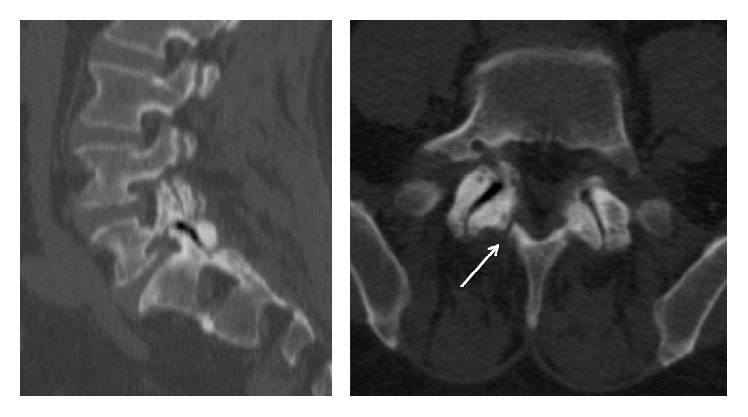
Preoperative CT. L5 interarticularis right pars spondylolysis (white arrow) and severe facet joint osteoarthritis with vacuum sign.

**Figure 4:**
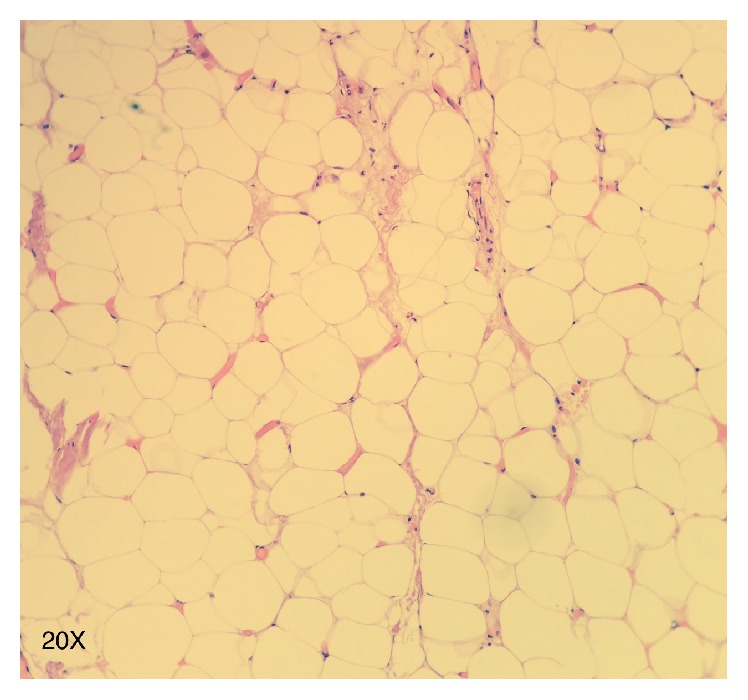
Histology. Hematoxylin-eosin stain (20x). Mature adipocytes and connective tissue cells with an elongated nucleus.

Here, we present the case of a 64-year-old male patient referred to our surgery unit for evaluation of lumbosacral pain and lower limb pain with episodes of neurogenic claudication. He had no history of steroid use, epidural steroid injections, or endocrinopathy. A physical examination revealed that he was overweight with a body mass index (BMI) of 32.5, and lumbosacral pain was observed by the negative bilateral straight leg raise test. No motor deficit was observed and the osteotendinous reflexes were preserved. Laboratory studies were performed to rule out endocrine disease, and normal values for thyroid, parathyroid, and adrenal hormones were found. MRI showed a deformity of the dural sac at the L4-S1 levels consistent with fatty tissue compression and signs of disc degeneration (Pfirrmann IV and Modic I) at the L4-L5 level (Figure 1). X-ray and CT showed L5-S1 instability secondary to low grade spondylolisthesis caused by spondylolysis and severe bilateral osteoarthritis facet joints (Figures 2 and 3). Initially, conservative treatment was recommended with weight loss, analgesics, and pregabalin for 6 months. Despite these recommendations and a weight loss of more than 10 kg, the lumbar and radicular left pain persisted with episodes of neurogenic claudication; thus, surgical treatment was performed. Decompressive laminectomy at the L4-L5 level with the removal of epidural fat was performed. Additionally, L4-S1 fusion was performed using pedicle screws, and interbody unilateral polyetheretherketone (PEEK) implants were placed using a transforaminal lumbar interbody fusion (TLIF) at the L5-S1 level. Bone graft for posterolateral spine fusion was obtained from posterosuperior iliac crest and the surgical field. Histopathological examination revealed mature adipose tissue that had grown in an infiltrative manner in absence of venous engorgement (Figure 4).

**Figure 5:**
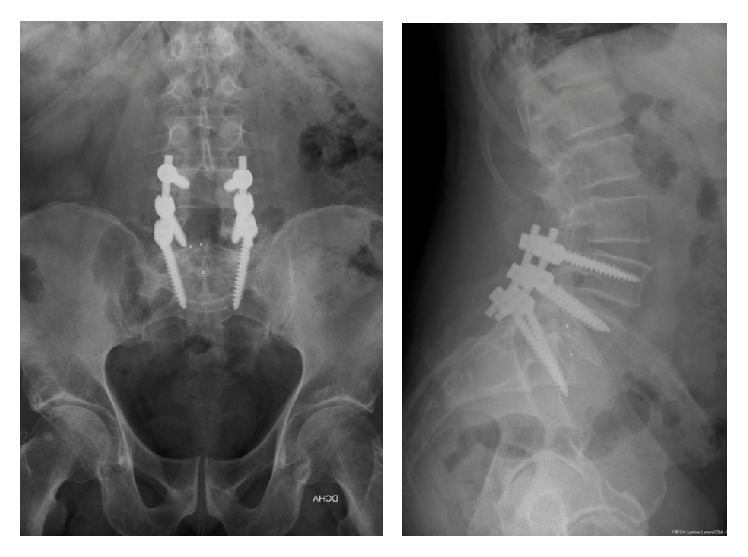
Postoperative X-ray. One-year follow-up.

At 6 months, the evolution of the disease examined clinically and radiologically was favourable. Disability and pain had improved after surgery. Postoperative X-ray showed signs consistent with probable radiographic fusion (Figure 5).

## 3. Discussion

Spinal epidural lipomatosis (SEL) is a rare condition. A literature review revealed 49 cases of idiopathic SEL and 62 cases of secondary SEL [1]. SEL is defined as a pathological overgrowth of normal fat tissue in the extradural space. Secondary SEL is often associated with chronic steroid use and endocrinopathies. Other reported secondary causes include adrenal tumours, hyperprolactinemia, and other endocrinopathies. Several years ago, there was talk of hypothyroidism as a cause of SEL, but this relationship is unclear [3–6]. Symptomatic epidural lipomatosis was first described in 1975 [2]. Idiopathic SEL has been shown to be associated with obesity [1, 7, 8] and many cases have been unrelated to any clear predisposing factors [1]. Badami and Hinck first reported idiopathic SEL in a woman with morbid obesity in 1982 [9], but it was not until 1991 that Haddad et al. coined the term idiopathic SEL [10].

A review of the literature revealed four categorical associations with SEL: exogenous steroid use, obesity, endogenous steroid excess or Cushing syndrome, and idiopathic causes [7]. Furthermore, a case of Paget disease related to SEL has been described [11], and the association of Scheuermann disease with thoracic SEL is common [12].

Epidural lipomatosis commonly presents with localized chronic pain that often lasts for several months to several years, followed by progressive or sudden neurological deficit. The most common spinal location of lipomatosis, regardless of its aetiology, is the thoracic region; this may be because this region contains more epidural fat [5, 13]. However, in a literature review, the incidence of thoracic and sacral involvement in SEL was similar [7]. It appears that when lipomatosis appears in the thoracic spine, it is more commonly related to previous steroid shots or a prior endocrine disease. When SEL affects the lumbosacral spine, as in our case, it is more commonly idiopathic [1]. In terms of surgery, the results were similar when the lumbosacral spine was compromised idiopathically or as secondary lipomatosis. However, in general, for SEL involving the thoracic spine, postoperative outcomes are worse for cases of secondary lipomatosis [1].

For thoracic SEL, exploration will reveal signs of spinal irritation, mainly ataxia, and may show signs and symptoms of myelopathy, which is the most frequently diagnosed cause of SEL. In lumbar SEL, there may be radicular impairment with neurogenic claudication.

Most case reports have used CT and myelography for SEL diagnosis [14, 15]. However, MRI is now the best test for the diagnosis of this condition. T1-weighted images differentiate epidural fat from dural content with a high degree of specificity. In our case, magnetic resonance imaging examination of the lumbosacral spine revealed spinal stenosis secondary to epidural lipomatosis extending from L4 to S1. This is termed the “Y” sign, which is a Y-shaped dural sac compressed by severe accumulation of epidural fat [16]. Borré et al. developed a classification for lumbosacral epidural lipomatosis by reviewing a series of 2,528 MR images. Lumbar epidural lipomatosis was classified into three stages based on the degree of lipomatosis seen on MRI in the axial plane. Patients with grade I disease were asymptomatic, while 17% of those with grade II disease were symptomatic. All patients with symptomatic grade III disease were believed to have severe stenosis. According to the grading scheme of Borré et al. our patient had grade III SEL [17].

MR allows the diagnosis of spinal epidural lipomatosis. However, in our case, L5-S1 slide in standing radiographs was suspected and axial and sagittal CT images showed the presence of spondylolysis. The routine evaluation of ventral instability in lumbar spondylolisthesis does not require dynamic radiographical examination if an upright lateral lumbar radiography and supine sagittal magnetic resonance imaging were done [18].

Electromyography is useful for determining the existence of acute or chronic radicular damage. Interestingly, Schulz et al. indirectly discovered an epidural lipomatosis case due to high impedance values observed during a failed spinal cord stimulator trial; this case was an old morbidly obese female with a history of chronic lower back and bilateral neuropathic leg pain encompassing the L3-S1 dermatomes. The authors suggest that this piece of information may have clinical application for subjects with obesity or a risk of endocrine disease, when high impedance is identified during a spinal cord stimulation trial with no obvious explanation [19].

First, an adequate anamnesis must be performed to identify any indication of lipomatosis of secondary origin. Laboratory tests are necessary to help rule out an endocrinopathy or another pathological conditions. The clinical presentation of epidural lipomatosis is the same as that of other compressive diseases. Epidural lipomatosis should be considered in the differential diagnosis of lumbar radiculopathy when there is an absence of common causes. CT or MR imaging is usually diagnostic for SEL.

The first-line treatment for idiopathic epidural lipomatosis has been weight loss and exercise therapy. Conservative therapy involves the discontinuation of steroid treatment and weight loss in those with exposure to exogenous steroids and in obese patients, respectively. Although weight reduction seems beneficial, this is applicable only for patients who present with mild symptoms but no significant signs [1]. Surgical intervention seems to be the treatment of choice when patients present with abnormal neurological signs and for patients who fail to respond clinically to a weight loss plan or are unable to lose weight. However, the failure of these measures could lead to surgical decompression with satisfactory results. Additionally, if degenerative changes or instability is present, decompression should be performed in association with a spinal fusion with instrumentation such as our case. Although the operative procedure itself is of low risk, postoperative treatment of comorbidities of the patient can be difficult [20]. Fessler et al. reported a 22% mortality rate in these patients after one year of follow-up [21]. Furthermore, a case of spontaneous resolution of epidural lipomatosis without the administration of any therapeutic measures has been described [22]. Our patient was an obese man, and other causes of epidural lipomatosis had been previously ruled; thus, weight loss was indicated. The patient lost at least 10 kg of weight; however, his neurological symptoms and mechanical lumbosacral pain due to instability at L5-S1 and degenerative disc disease at L4-L5 persisted; therefore, surgery was indicated.

The surgery was performed by wide decompressive laminectomy at the L4-L5 level with the removal of epidural fat. L4-S1 fusion was performed using pedicle screws, and interbody unilateral PEEK implants were placed by transforaminal approach at L5-S1. Fusion of L4-L5 level was considered by the wide decompression (partial bilateral facetectomy) and degenerative disc changes (Pfirrmann IV and type I Modic changes) [8, 23, 24]. Additionally, bone graft obtained from the posterosuperior iliac crest and bone from the surgical field were placed in the field.

In conclusion, spinal epidural lipomatosis is more common in patients on corticosteroid treatment for long periods of time, but it can have other causes. Conservative therapy, either weight loss or withdrawal of steroids, may reduce epidural adipose tissue leading to an improvement of symptoms. Decompressive laminectomy with resection of the epidural fat is also very successful in improving neurological symptoms. Additionally, if there are degenerative changes or instability, spinal fusion is justified.
